# Retraction: Receptor-Recognized α2-Macroglobulin Binds to Cell Surface-Associated GRP78 and Activates mTORC1 and mTORC2 Signaling in Prostate Cancer Cells

**DOI:** 10.1371/journal.pone.0325675

**Published:** 2025-06-05

**Authors:** The PLOS One Editors

After this article [1] was published, concerns were raised regarding results presented in Figs 2-4, Fig 6, and Figs 8-10.

Specifically,

- The following panels in this article [1] appear similar despite representing different experimental conditions:
  - Fig 2A Controls mTOR and Fig 2B Controls Rheb.
  - Fig 2A Controls TSC2, Fig 2B p-TSC2, and Fig 3A 4EBP1 control.
  - Fig 3A p-S6K and Fig 3B p-S6K.
  - Fig 6B IB p-S6K and Fig 6B IB p-4EBP1.
  - Fig 6B IB Actin and Fig 8A Akt
- The following panels presented in this article [1] appear similar to panels published in [2–13] despite representing different experimental conditions:
  - Partial or full panel similarity between Fig 2A Controls Raptor in [1], the Fig 8G S6K in [2], Fig 1B COX-2 control for DU-145 in [3, retracted in 4], and Fig 5C GADD45β in [5].
  - Fig 2A p-TSC2 in [1], Fig 4 COX-2 control for PC-3 in [3, retracted in 4], Fig 4A actin in [5], Fig 4B GADPH in [5], Fig 4B FOXO1 in [5], Fig 5C in [6], and Fig 1C CREB in [7].
  - Fig 2B Raptor in [1], Fig 2B GβL in [1], and Fig 5A DU145 actin in [8].
  - Fig 4A Akt control in [1] and Fig 5 1-LN actin for TFII-I in [9].
  - Fig 4B p-Akt^S473^ in [1], Fig 2A p-PERK in [10], and Fig 2C p-elF2α in [10].
  - Fig 4B Akt control in [1] and Fig 2D elF2α in [10].
  - Fig 6B IB GRP78 in [1] and Fig 2B DU-145 in [11, retracted in 12].
  - Fig 6C IB p-4EBP1 in [1] and Fig 8C 1-LN Rictor in [3, retracted in 4].
  - Fig 8C Akt in [1], Fig 8D (lower panel) in [1], Fig 1A 1-LN actin for Bak in [9], Fig 7 DU-145 control in [3, retracted in 4], and Fig 7 1-LN Actin in [3, retracted in 4].
  - Fig 8C Akt^S473^ in [1], Fig 2A Actin in [11, retracted in 12], Fig 4A p-Akt^S473^ in [13], and Fig 7B p-PRAS in [13].
  - Fig 10 Insulin dsRictor RNAi pAkt^S473^ in [1] and Fig 2B 1-LN in [11], retracted in 12].
- There appear to be inconsistencies between the splice lines indicated for the following paired results, suggesting that the protein and corresponding control results were not obtained from the same blot and cannot not be compared directly.
  - Fig 9C p-Akt^S473^ and Akt
  - Fig 10 Insulin dsRaptor RNAi p-Akt^T308^ and Insulin dsRaptor RNAi p-Akt^S473^

During editorial follow-up, raw blot data underlying most of the panels of concern were provided, however these data were insufficient to resolve most of the concerns listed above and raised concerns for the labelling, storage, and reliability of the data underlying the results presented in this article.

In light of the above unresolved concerns that question the integrity and reliability of the reported results and conclusions, the *PLOS One* Editors retract this article.

SVP did not respond to the final editorial decision. UKM either did not respond directly or could not be reached.

The following panels present results that appear similar to material previously published in [8–10] and [13] which are not offered under a CC BY license and are therefore excluded from this article’s [1] license:

- The Fig 2B Raptor and GβL panels in [1] which report results similar to material previously presented in [8] published in 2009 by American Association for Cancer Research.
- The Fig 4A Akt control panel, the Fig 8C Akt panel, and Fig 8D lower panel in [1] which report results similar to material previously presented in [9] published in 2011 by Wiley.
- The Fig 4B p-Akt^S473^ and Akt control panels in [1] which report results similar to material previously presented in [10] published in 2005 by Society for Leukocyte Biology.
- The Fig 8C Akt^S473^ panel which report results similar to material previously presented in [13] published in 2012 by Wiley.
